# Identification of Biomarkers of Arrhythmogenic Cardiomyopathy (ACM) by Plasma Proteomics

**DOI:** 10.3390/medicina61010105

**Published:** 2025-01-13

**Authors:** Sinda Zarrouk, Houda Ben-Miled, Nadia Rahali, Josef Finsterer, Fatma Ouarda

**Affiliations:** 1 Technological Platform IPTOMICS, Pasteur Institute of Tunis, University of Tunis El Manar, Tunis 1002, Tunisia; 2 LR99ES10 Human Genetics Laboratory, Faculty of Medicine of Tunis, University of Tunis El Manar, Tunis 1002, Tunisia; 3 Biochemistry and Biotechnology Laboratory LR01ES05, Faculty of Sciences of Tunis, University of Tunis El Manar, Tunis 1002, Tunisia; 4 Neurology Department, Neurology & Neurophysiology Center, 1180 Vienna, Austria; 5 Service de Cardiologie Pédiatrique, Hôpital la Rabta Tunis, Tunis 1007, Tunisia

**Keywords:** arrhythmogenic cardiomyopathy, biomarkers, proteomics, desmosomal proteins, phosphorylation

## Abstract

*Background and Objectives:* The pathophysiology of arrhythmogenic cardiomyopathy (ACM), previously known as arrhythmogenic right ventricular cardiomyopathy (ARVC), and its specific biological features remain poorly understood. High-throughput plasma proteomic profiling, a powerful tool for gaining insights into disease pathophysiology at the systems biology level, has not been used to study ACM. This study aimed at characterizing plasmatic protein changes in patients with ACM, which were compared with those of healthy controls, and at exploring the potential role of the identified proteins as biomarkers for diagnosis and monitoring. *Materials and Methods:* Blood samples were collected from six ACM patients, four patients with other cardiomyopathies, and two healthy controls. Plasma was processed to remove high-abundance proteins and analyzed by two-dimensional gel electrophoresis. Differential protein expressions were assessed using PDQuest software, Bio-Rad US version 8.0.1. *Results:* The analysis revealed several proteins with altered expressions between ACM patients and controls, including plakophilin-2, junctional plakoglobin, desmoplakin, desmin, transmembrane protein 43, and lamin A/C. *Conclusions:* The plasma proteomic profiling of ACM suggests that ACM is a distinct disease entity characterized by a unique dysregulation of desmosomal proteins. The identification of plasma biomarkers associated with ACM underscores their potential to improve diagnostic accuracy and facilitate early intervention strategies. Further exploration of mutations in desmosomal proteins and their phosphorylation states may provide deeper insights into the pathophysiology of ACM.

## 1. Introduction

Arrhythmogenic right ventricular cardiomyopathy (ARVC) is a genetic cardiomyopathy originally believed to involve only the right ventricle. Since then, there has been increasing evidence that there are also left-predominant and biventricular forms of ARVC [1]. Therefore, ARVC was recently renamed arrhythmogenic cardiomyopathy (ACM) by the Heart Rhythm Society (HRS) and the European Heart Rhythm Association (EHRA) [2]. Two theories explain the progressive fibro-fatty replacement of the myocardium: (1) inflammation in response to myocardial injury [3], which leads to myocyte death by apoptosis or necrosis, although it is unclear whether inflammation is a primary or secondary event; (2) apoptosis following rupture of the interstitial disk [4,5], which causes electromechanical instability and leads to ventricular arrhythmias. In addition, mutations in desmosomal genes such as plakophilin-2 (*PKP2*) are thought to cause the infiltration of fibroblasts and adipocytes into the myocardium [6], which progresses from the epicardium to the endocardium. Despite advances in understanding the pathophysiology of ACM, the mechanisms remain complex, and several other theories have been proposed.

Human genetic studies have shown that 40–50% of patients with ACM carry mutations in desmosomal genes that are crucial for cell adhesion in tissues but are exposed to permanent mechanical stress, such as in the heart muscle [7]. Desmosomes, together with fascia adhesion and gap junctions, coordinate muscle contraction and electrical coupling in the heart [8]. A mutation in a single desmosomal gene can cause a “domino effect” leading to the loss of expression in other desmosomal and gap junction proteins [9]. This disruption leads to structural and functional changes, including the abnormal distribution of ion channels, junctional remodeling, electrical abnormalities, and the dysregulation of mitochondrial and calcium processes [10].

Intercellular junctions occupy a central region for adhesion and a regional plate for the attachment of intermembrane filaments in myocytes. Three groups of desmosomal proteins have been identified: (1) transmembrane desmosomal cadherins such as desmocollin 2 and desmoglein 2 (DSG2); (2) desmoplakin (DSP), which binds to intermediate filaments (desmin in cardiac muscle); and (3) binding proteins such as JUP (catenin-γ) and PKP2, which link the desmosomal cadherins and DSP. About half of ACM patients have mutations in these genes [11]. In addition, rare non-desmosomal mutations have been identified in genes involved in functions such as cytoskeletal architecture, calcium management, sodium transport, and cytokine signaling [12,13].

Cytoskeletal defects can disrupt the structural integrity and mechanotransduction of cardiomyocytes in ACM [14]. Novel mutations in genes such as *DES* (desmin), *LMNA* (lamin A), *TMEM43* (transmembrane protein 43), *TTN* (titin), and *FLNC* (filamin C) were found in ACM patients by gene sequencing. Desmin, an intermediate filament, connects sarcomeric Z-discs to the sarcolemma, desmosomes, and nuclear envelope [15]. The LINC complex connects the nuclear envelope to the cytoskeleton, with key components such as A- and B-type lamins involved in chromatin organization, DNA replication, and gene expression [16]. Transmembrane protein 43 (LUMA) associates with lamins and is involved in nuclear membrane organization [17]. The *TMEM43* mutation p.S358L was identified as a disease-causing mutation in a family from Newfoundland, Canada [18].

Calcium homeostasis is essential for cardiomyocyte function as it regulates excitation-contraction coupling and influences the electrophysiology of the heart. A disturbed calcium balance can lead to cardiac arrhythmias by causing abnormal depolarizations. Mutations in genes involved in calcium regulation have been linked to ACM, which could be a possible mechanism in arrhythmogenesis. Ryanodine receptor 2 (RYR2) plays a key role in calcium release from the sarcoplasmic reticulum and triggers calcium transients that initiate sarcomere contraction [19].

The profibrotic cytokine transforming growth factor-β3 (TGFβ3 encoded by TGFB3) has also been associated with various forms of ACM. TGFB3—particularly the locus 14q24.3—is an attractive candidate gene given the pro-inflammatory and profibrotic activities of TGFβ3. However, sequencing did not identify any variants in the coding region that could be linked to ACM [12,20].

Protein modifications change the physico-chemical properties of proteins through chemical and biochemical reactions and thus influence their function. These modifications can activate or inhibit proteins, stabilize them, promote their degradation, change their interaction partners, or influence their cellular localization. Typically, modifications lead to structural changes by altering the charge or hydrophobicity of target residues, which can lead to conformational shifts and affect active sites, interaction domains, or ligand binding. A single gene can produce different protein forms due to genetic variations, transcriptional differences, alternative splicing, alternative translation initiation, or post-translational modifications [21].

The development of clinical proteomic biomarkers is an emerging and rapidly growing area in human biomedical research. Over the past decade, proteomic studies on human plasma and other biofluids have made significant progress, enabling accurate quantification of proteins and potential biomarkers with increasing depth and coverage [22].

Thus, the proteomic approach enables the reliable identification of biomarkers for diagnostic and prognostic purposes in different types of diseases. Two-dimensional electrophoresis (2-DE) is the most commonly used proteomic tool in research, as it allows not only the comparison of protein quantities but also of their isoforms on the same gel. This technique is based on two basic principles: firstly, the separation of proteins according to their isoelectric point, and secondly, two-dimensional sodium dodecyl sulphate polyacrylamide gel electrophoresis (SDS-PAGE), which separates proteins according to their MW [23]. In the context of cardiovascular health and disease, proteomics plays a critical role in identifying the pathogenesis and progression of various conditions, including cardiac developmental defects, atherosclerosis, hypertension, myocarditis, cardiomyopathies, myocardial infarction, arrhythmias, heart failure, aneurysms, and stroke [24].

Plasma is biochemically the most valuable clinical sample as it contains the largest and most comprehensive type of human proteome. These characteristics make it the most difficult sample to process in proteomics, despite the relatively favorable behavior (i.e., solubility) of its components. The enormous size of the plasma proteome reflects the sheer number of different proteins that need to be detected.

The aim of the following study was to use 2-DE to detect one or more sensitive and specific biomarkers for ACM in the plasmatic proteome of ACM patients.

## 2. Materials and Methods

### 2.1. Sample Collection and Processing

Blood samples were collected from six patients (two men and four women) from five families diagnosed with ACM. These families were recruited from the Department of Cardiology at La Rabta Public Teaching Hospital in Tunis, Tunisia. The clinical study involved comprehensive assessments through transthoracic echocardiography and cardiac magnetic resonance imaging (MRI). Informed consent was obtained from all participants, including six family members and two unrelated healthy controls, who resided in the same region.

To prepare plasma, 10 mL of blood was collected and mixed with 1.7 mg of potassium EDTA as an anticoagulant immediately after venipuncture to prevent clotting. The blood samples were then centrifuged at 3000 rpm for 10 min at 25 °C to separate the plasma from the cellular components. The resulting plasma was stored at −20 °C for further analysis.

### 2.2. Depletion of High-Abundance Plasma Proteins

Because albumin and immunoglobulin IgG collectively account for ~76% of the total plasma protein content, we selectively removed these proteins to enrich for proteins of lower abundance. A dye-based proteoprep blue albumin and IgG depletion kit (Sigma Aldrich, Berlin, Germany) was used according to the manufacturer’s instructions. Briefly, the suspended, slurry medium was added to the spin columns, centrifuged, and equilibrated at 8000× *g* for 10 s. The spin columns were collected in fresh collection tubes. To each spin column, 0.1 mL of plasma sample was added to the packed medium bed; they were incubated for 10 min, centrifuged at 8000× *g* for 60 s, and the same step was repeated twice to remove the additional albumin. The two-times-depleted plasma remained in the collection tube and was pooled for optimal protein recovery. The albumin-/IgG-depleted plasma samples were stored at −80 °C for long-term storage. Each trial was repeated independently in triplicate.

### 2.3. SDS-PAGE Analysis

The total protein content in the plasma samples was determined using a BCA kit (Bicinchoninic Acid Assay, Sigma, Lausanne, Suisse), for which Bovine Serum Albumin (BSA) was used. A volume of the sample was dissolved in a volume of denaturant buffer (62.5 mM Trise HCl pH 6.8, 20% *v*/*v* glycerol, 2% *w*/*v* SDS, 5% β-mercaptoethanol, 1% *w*/*v* bromophenol blue) and was boiled for 5 min at a temperature of 100 °C. Then 1.6 microliters (20 μg) of albumin- and IgG-depleted proteins were separated under reducing conditions on 8% SDS-PAGE mini gels (7.2 cm × 8.6 cm) at 40 V for 15 min and constant currents of 60 V for 2 h and visualized by silver staining according to standard protocols. The gels were scanned in a GS-800™ calibrated densitometer Bio-Rad, Berlin, Germany. Each trial was repeated independently in triplicate.

### 2.4. One-Dimensional IEF Using the Protean IEF Cell

Protein samples were dissolved in 125 μL of rehydration buffer containing 7 M urea, 2 M Thiourée, 4% *w*/*v* CHAPS, 50 mM DTT, 0.2% *v*/*v* pH 3–10 and pH 5–8 ampholyte, and 0.001% bromophenol blue. Gel strips 7-cm-long with linear pHs of 3–10 and gel strips 7-cm-long with linear pHs of 5–8 (Bio-Rad, Hercules, CA, USA) were placed in a rehydration buffer with samples in a Protein IEF Cell Bio-Rad machine, Berlin, Germany for 16 h of passive rehydration. After rehydration, the focusing tray was renewed to remove any proteins not absorbed into the strip. IEF was conducted using a Protean IEF Cell (Bio-Rad) according to the following IEF parameters: 250 V for 15 min, 4000 V for 2 h of linear ramping, and 4000 V for 12 kVh. Each trial was repeated independently in triplicate.

### 2.5. Two-Dimensional SDS-PAGE

For the 2DE analysis, individual samples (n = 8) were analyzed at least three times. Prior to SDS-PAGE, the IPG strips were equilibrated twice for 15 min with gentle shaking. The first equilibration solution contained 0.375 M of Tris-HCl, pH 8.8, 6 M urea, 20% *v*/*v* glycerol, 2% *w*/*v* SDS, 130 mM DTT, and 0.01% *w*/*v* BPB. In the second equilibration solution, DTT was replaced by 135 mM iodoacetamide. The equilibrated IPG strips were lightly rinsed with milli-Q water, blotted to remove excess equilibration buffer, and then mounted on SDSPAGE gels (7.2 cm × 8.6 cm × 1 mm), 8% polyacrylamide (30% (*w*/*v*), acrylamide: 0.8% ((*w*/*v*) bis-acrylamide) using a Mini PROTEAN tetracell system (Bio Rad, Paris, France) at 40 V for 10 min, followed by 60 V for 150 min until the dye was removed off the edge of the 2D gel.

### 2.6. Protein Visualization

Using the silver staining method, the gels were fixed for 1 h in 50% *v*/*v* ethanol and 50% *v*/*v* acetic acid. The gels were then incubated in 5% ethanol, 5% acetic acid overnight. The gels were rinsed with milli-Q water for 1 h then again fixed with 1% *v*/*v* glutaraldehyde, 0.5 M sodium acetate for 45 min. The fixed gels were rinsed with milli-Q water three times for 15 min each. Gels were immersed in 1.5% *w*/*v* silver nitrate, 0.2% *w*/*v* sodium hydroxide, and 1.3% *v*/*v* ammonium hydroxide for 1 min and rinsed with milli-Q water three times. It was developed with 0.1% formaldehyde and 0.01% *w*/*v* citric acid. Finally, the reaction was stopped with 2% *v*/*v* acetic acid. The stained gels were scanned with a densitometry (Bio-Rad, Hercules, CA, USA) GS-800 for analysis. Subsequently, the presence or absence and the value of each protein spot were analyzed using PD-Quest and Quantity One (Bio-Rad, Paris, France).

## 3. Results

**Figure 1:**
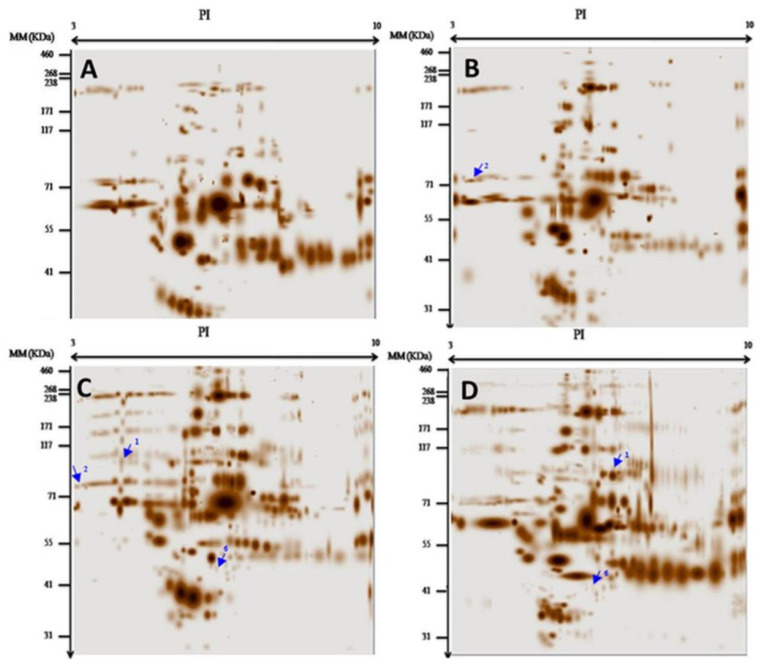
Protein profiles obtained by 2DE pH 3-10. The figures (**A**–**D**) are for the gel proteins profiles (**A**) Gel A Protein profile of female control (TF), (**B**) Gel B Protein profile of patient A2, the arrow 2 represent LMNA, (**C**) Gel C Protein profiles of patient A8, the arrow 1 represented PKP2; the arrow 2 represent LMNA and the arrow 6 represent TMEM43, (**D**) Gel D Protein profiles of patient A9; the arrow 1 represent PKP2 and the arrow 6 represent TMEM43). The arrow numbers 1, 2, 3, 4, 5, and 6 represent the proteins: 1: PKP2, 2: LMNA, 3: DSP, 4: JUP, 5: DES, 6: TMEM43. The X-axis stands for the isoelectric point (PI), and the Y-axis for the molecular mass (MM).

**Figure 2:**
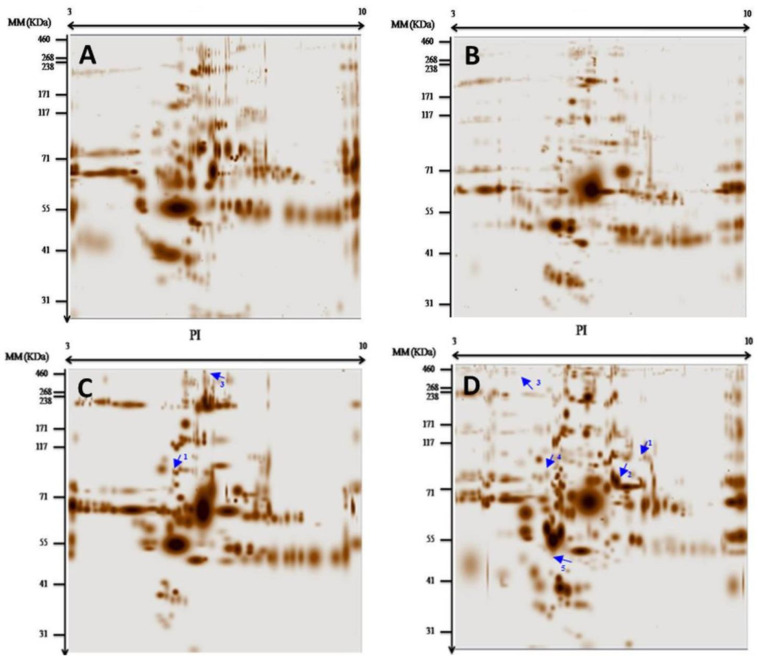
Protein profiles obtained by 2DE pH 3–10. The figures (**A**–**D**) are for the gel proteins profiles: (**A**) Gel E Protein profile patient A12, (**B**) Gel F Protein profile male control, (**C**) Gel G Protein profiles of patient A11, the arrow 1 represented PKP2; the arrow 3 represent DSP, (**D**) Gel H Protein profiles of patient A13; the arrow 1 represent PKP2, the arrow 2 represent LMNA the arrow 3 represent DSP, the arrow 4 represent JUP and the arrow 5 represent DES. The arrow numbers 1, 2, 3, 4, 5, and 6 are for the proteins: 1: PKP2, 2: LMNA, 3: DSP. The X-axis stands for the isoelectric point (PI), and the Y-axis for the molecular mass (MM).

In this study, we used a differential proteomic approach to identify specific biomarkers for ACM in the plasma proteome of patients compared to healthy and diseased controls. Taking advantage of the high resolving power of the 2DE, we characterized protein spots in a pH range of 3–10. Qualitative and quantitative analyses performed with PDQuest software version 8.0.1 (Bio-Rad, USA) revealed an average of 100 protein spots with differential expressions between patients and controls (Figure 1). These variations were categorized into four different types: 1. protein spots presented in the patients but not in the controls; 2. identical protein spots in two or more patients with different intensities (Figure 1C,D and Figure 2A); 3. protein spots found in the controls but not in the patients (Figure 1A,C,D and Figure 2A,B); and 4. spots common to both groups but with different intensities. A comparison of patient and control gels revealed the presence of protein spots corresponding to PKP2, JUP, DSP, DES, TMEM43, and LMNA, each with unique isoelectric points (pIs). In particular, the spots for DSC2, DSG2, and TGFβ3 were not detected.

The PKP2 protein with a molecular weight (MW) of 97.41 kDa and a predicted pI of 9.39 in the unphosphorylated state was identified in the plasma of patients A8 (Figure 1C), A9 (Figure 1D), A11 (Figure 2C), and A13 (Figure 2D) (Table 1). We focused on the phosphorylation state and observed pI values of 4.21, 6.23, 5.16, and 6.87 for these patients, respectively. Using the PhosphoSitePlus database, we found that the number of phosphorylations in these patients was 94, 30, 56, and 19, respectively. The absence of PKP2 in healthy controls underscores its role as a disease-specific marker, with lower pI values correlating with higher phosphorylation levels. The distinct phosphorylation profile of PKP2 thus makes it a promising biomarker for ACM that could enable early diagnosis and better treatment of patients.

Similarly, we analyzed the JUP protein, which has a molecular mass of 81.7 kDa and a theoretical pI of 5.75. In patient A13, a stain corresponding to JUP was detected (Figure 2D) with a pI of 4.95, indicating phosphorylation. This change was not observed in the healthy controls, further emphasizing the potential of JUP as a disease-specific biomarker. Importantly, mutations in the JUP gene are documented in the Proteome Scout database as confirmed pathologic mutations associated with ACM, underscoring its importance. The distinct phosphorylation profile of JUP in patient A13 suggests that it warrants further investigation as a plasma biomarker to expand our understanding of ACM mechanisms and improve diagnostic capabilities.

The DSP protein with a molecular mass of 331.77 kDa and a theoretical pI of 6.44 was analyzed in patients A11 (Figure 2C) and A13 (Figure 2D). The pI values were 5.96 for A11 (17 phosphorylation sites) and 4.60 for A13 (187–188 phosphorylation sites), whereas neither site was detected in the healthy controls. This significant difference in phosphorylation levels underscores the importance of DSP in ACM. The unique phosphorylation profiles of DSP in both patients position it as a potential plasma biomarker that contributes to our understanding of the underlying disease mechanism and improves diagnostic strategies.

In our analysis of the DES protein, which has a MW of 53.53 kDa and a theoretical pI of 5.21, we found a patch with a pI of 5.08 associated with three phosphorylation sites in patient A13. This modification was not present in the healthy controls, indicating its potential as a disease-related biomarker. Of note, mutations in the DES gene were also associated with ACM, as recorded in the Proteome Scout database. The phosphorylation profile of DES in patient A13 highlights its potential role in improving the diagnostic accuracy of ACM.

The LMNA protein with a MW of 74.13 kDa and a theoretical pI value of 6.57 was identified in the plasmas of patients A2, A8, and A13. Each patient had different pI values: 3.37 for 112 phosphorylations in A2, 3.07 for 124 phosphorylations in A8, and 6.42 for 1 phosphorylation in A13. The different phosphorylation profiles of LMNA suggest its potential utility as a plasma biomarker for ACM and provide further insight into the mechanisms of the disease.

The TMEM43 protein with a MW of 44.87 kDa and a theoretical pI of 7.86 was analyzed in patients A8 (Figure 1C) and A9. Both showed TMEM43 spots with different pI values: 5.73 for A8 (10 phosphorylation sites) and 6.03 for A9 (7 phosphorylation sites). The absence of these spots in the healthy controls emphasizes the specific changes associated with ACM. The phosphorylation profiles of TMEM43 underpin its potential as a plasma biomarker to help understand the pathophysiology of the disease and potentially aid in diagnosis and monitoring.

## 4. Discussion

The identification of PKP2, JUP, DSP, DES, LMNA, TMEM43, and their phosphorylation states in the plasma of ACM patients suggests that they may serve as biomarkers for early diagnosis and monitoring and improve our understanding of the underlying mechanisms of this disease. The discovery of these specific biomarkers in the plasma of ACM patients provides crucial insights into the pathophysiology of the disease and opens new avenues for diagnosis and therapeutic strategies. The use of 2DE electrophoresis in this study shows that proteins such as PKP2, JUP, DSP, DES, LMNA, and TMEM43 are consistently present in the plasma of ACM patients, whereas they are absent in healthy and diseased controls. This specificity could improve diagnostic accuracy and facilitate early intervention strategies.

The consistent absence of these biomarkers in healthy individuals underscores their potential as disease-specific indicators. The identified phosphorylation states of these proteins provide insight into the pathophysiological processes underlying ACM. For example, the different phosphorylation profiles of PKP2 and other desmosomal proteins suggest that aberrant phosphorylation may play a crucial role in the pathophysiology of ACM. Previous studies have shown that the altered phosphorylation of desmosomal proteins can disrupt cell–cell adhesion and lead to cardiomyocyte dysfunction [25,26]. The correlation between these phosphorylation changes and ACM pathology may represent a new area for therapeutic intervention.

Furthermore, the absence of these proteins in plasma samples from patients with hCMP, dCMP, and LVNC underlines their potential specificity for ACM. This observation is consistent with the results of other studies that have found different molecular profiles in different cardiomyopathies [27]. By comparing the biomarker profiles in these diseases, we can better differentiate ACM from other forms of cardiomyopathy, potentially leading to tailored treatment approaches.

Our results also emphasize the importance of genetic mutations within the desmosome complex in the context of ACM. Mutations in genes such as *PKP2, DSP*, and *JUP* are well-documented and contribute to the pathogenesis of ACM [28,29]. The desmosome complex is critical for maintaining the mechanical integrity of cardiac tissue, and mutations can lead to impaired cell adhesion, which in turn leads to myocardial disorganization and electrical instability.

The integration of genetic screening for these mutations in conjunction with the analysis of plasma biomarkers could significantly improve our understanding of individual risk profiles in ACM. For example, a patient with a known mutation in the PKP2 gene who also has elevated levels of phosphorylated PKP2 in plasma could be at an increased risk of developing severe ACM. This dual approach of gene and biomarker analysis could be crucial for the development of prevention strategies and personalized treatment plans.

Differences in protein phosphorylation states between patients, despite identical clinical diagnoses of ACM, can be attributed to several factors. Firstly, phosphorylation is a dynamic process that can vary according to various biological parameters, such as individual gene expression, variations in cellular signaling pathways, or interactions with other proteins [30]. In addition, differences in patients’ biological environments, such as the influence of drugs, nutritional factors, or comorbidities, can also modulate the protein phosphorylation status. Furthermore, the heterogeneity of the disease itself, even under the same clinical diagnosis, can result in distinct biological profiles between patients, reflecting varied underlying mechanisms influencing protein phosphorylation [30,31,32]. These factors combined explain why different phosphorylation states can be observed for the same protein in patients clinically diagnosed with ACM.

In addition, further studies are needed to elucidate the role of protein kinases in the phosphorylation of desmosomal proteins. Kinases such as PKC (protein kinase C) and PKA (protein kinase A) are involved in the phosphorylation of desmosomal proteins, which can modulate their function and stability [33]. Understanding the action of specific kinases involved in the phosphorylation of the proteins identified in our study could provide valuable insights into the molecular mechanisms underlying ACM. The pharmacological treatment of these kinases could open new therapeutic options for the treatment of ACM.

Future research should aim to investigate the mechanistic relationships between identified plasma biomarkers, their phosphorylation states, and the underlying genetic mutations in ACM patients. Longitudinal studies examining changes in these biomarkers over time could help elucidate their role in disease progression. Furthermore, expanding the study to a larger cohort of patients from diverse backgrounds could confirm the specificity of these biomarkers. Furthermore, the use of advanced proteomic techniques such as mass spectrometry could reveal additional biomarkers that contribute to the pathogenesis of ACM. The integration of multi-omic approaches, including genomics, proteomics and metabolomics, could provide a holistic understanding of ACM and its molecular basis.

## 5. Conclusions

In summary, the identification of plasma biomarkers in ACM represents an exciting development in cardiology and offers opportunities for improved diagnosis and personalized treatment. By focusing on the absence of these biomarkers in healthy controls and their specific alterations in ACM, as well as considering the genetic landscape and the role of protein kinases, we can pave the way for innovative research and clinical applications. Our study also aimed to emphasize that the results obtained primarily serve to lay the foundation for an innovative diagnostic approach. Future studies, with a larger number of patients, will help validate and further explore these results and conclusions.

As perspectives for this study, mass spectrometry plays a crucial role in confirming the identity of proteins separated by 2DE, providing detailed information about their mass and structure. This complementarity allows for the precise validation of the identified plasma biomarkers. By combining 2DE with mass spectrometry, it is possible not only to confirm the presence of these biomarkers but also to verify their specificity, thereby enhancing the reliability of the results and enabling a more accurate diagnosis.

## Figures and Tables

**Table 1:** Results of analysis plasma of subjects affected with ACM by 2DE.

Protein	Genes	Chromosomal Location	Reference UniprotKB	Number of Amino Acids and Type of Isoform	Molecular Weight (KDa)	Theoretical pI Unphosphorylated State	pI		Number of Phosphorylated Residues
							Patient	pI Phosphorylated State	
Plakophiline 2	*PKP2*	12p11	Q99959	881 Isoform 2	97.41	9.39	A8	4.21	94
							A9	6.23	30
							A11	5.16	56
							A13	6.87	19
Lamine A/C	*LMNA*	1q22	P02545	664 Isoform A	74.13	6.57	A2	3.37	112
							A8	3.07	124
							A13	6.42	1
LUMA (TMEM43)	*TMEM43*	3p25.1	Q9BTV4	400	44.87	7.86	A8	5.73	10
							A9	6.03	7
Desmoplakine	*DSP*	6p24	P15924	2871	331.77	6.44	A11	5.96	17
							A13	4.60	187 188
Plakoglobine	*JUP*	17q21	P14923	145	81.74	5.75	A13	4.95	17
Desmine	*DES*	2q35	P17661	470	53.53	5.21	A13	5.08	3

## Data Availability

All data are available from Sinda Zarrouk.
